# Supporting Life Adjustment in Patients With Lung Cancer Through a Comprehensive Care Program: Protocol for a Controlled Before-and-After Trial

**DOI:** 10.2196/54707

**Published:** 2024-02-13

**Authors:** Wonyoung Jung, Alice Ahn, Genehee Lee, Sunga Kong, Danbee Kang, Dongok Lee, Tae Eun Kim, Young Mog Shim, Hong Kwan Kim, Jongho Cho, Juhee Cho, Dong Wook Shin

**Affiliations:** 1 Department of Family Medicine and Supportive Care Center Samsung Medical Center Sungkyunkwan University Seoul Republic of Korea; 2 Department of Medicine Sungkyunkwan University School of Medicine Seoul Republic of Korea; 3 Patient-Centered Outcomes Research Institute Samsung Medical Center Seoul Republic of Korea; 4 Department of Psychology and Philosophy Sam Houston State University Huntsville, TX United States; 5 Department of Clinical Research Design and Evaluation Samsung Advanced Institute for Health Science & Technology Sungkyunkwan University School of Medicine Seoul Republic of Korea; 6 Center for Clinical Epidemiology Samsung Medical Center Seoul Republic of Korea; 7 Department of Thoracic and Cardiovascular Surgery Samsung Medical Center Sungkyunkwan University School of Medicine Seoul Republic of Korea

**Keywords:** comprehensive care, early intervention, adjustment to cancer, return to work, lung cancer, unmet needs, lung, lungs, pulmonary, respiratory, cancer, oncology, prehabilitation, survivor, survivors, survivorship, education, educational

## Abstract

**Background:**

Lung cancer diagnosis affects an individual’s quality of life as well as physical and emotional functioning. Information on survivorship care tends to be introduced at the end of treatment, but early intervention may affect posttreatment adjustment. However, to the best of our knowledge, no study has explored the effect of early information intervention on the return to work, family, and societal roles of lung cancer survivors.

**Objective:**

We report the study protocol of a comprehensive care prehabilitation intervention designed to facilitate lung cancer survivors’ psychological adjustment after treatment.

**Methods:**

A comprehensive care program was developed based on a literature review and a qualitative study of patients with lung cancer and health professionals. The Lung Cancer Comprehensive Care Program consists of educational videos and follow-up visits by a family medicine physician. To prevent contamination, the control group received routine education, whereas the intervention group received routine care and intervention. Both groups completed questionnaires before surgery (T0) and at 1-month (T1), 6-month (T2), and 1-year (T3) follow-up visits after surgery. The primary outcome was survivors’ psychological adjustment to cancer 6 months after pulmonary resection.

**Results:**

The historical control group (n=441) was recruited from September 8, 2021, to April 20, 2022, and the intervention group (n=350) was recruited from April 22, 2022, to October 17, 2022. All statistical analyses will be performed upon completion of the study.

**Conclusions:**

This study examined the effectiveness of an intervention that provided general and tailored informational support to lung cancer survivors, ranging from before to the end of treatment.

**Trial Registration:**

ClinicalTrials.gov NCT05078918; https://clinicaltrials.gov/ct2/show/NCT05078918

**International Registered Report Identifier (IRRID):**

DERR1-10.2196/54707

## Introduction

Lung cancer remains the leading cause of cancer-related mortality worldwide and is prevalent in Korea [1,2]. However, advancements in screening and treatment have notably improved lung cancer outcomes. For example, the 5-year conditional recurrence-free survival rate of patients with surgically resected non–small cell lung cancer (NSCLC) in Korea increased from 51.5% to 68.5% in those diagnosed in the 1990s and the 2010s, respectively [3]. Consequently, the growing number of lung cancer survivors underscores the urgent need to address posttreatment needs.

Surviving lung cancer can substantially impact the quality of life and emotional health [4-6]. Many survivors adjust over time; however, persistent fear and the expectation of a rapid return to normal posttreatment life are common [7,8]. While initial postdiagnosis support is robust, survivors often encounter a new spectrum of concerns as they transition to life after cancer, including managing lingering symptoms and comorbidities that can affect overall well-being [8-10]. The commonly reported symptoms among lung cancer survivors include fatigue, pain, dyspnea, and impaired pulmonary function [11-13]. They also experience psychological distress and a fear of cancer recurrence [14,15]. Studies have shown that interventions focusing on physical rehabilitation and psychological adaptations can be effective for cancer survivors [16,17]. These interventions address both the physical and emotional challenges faced during the recovery process.

Despite the abundant information and support available after diagnosis, survivors later find themselves having a different set of questions and concerns related to life after cancer [18]. Our prior qualitative study of patients with lung cancer revealed the need for information on the expected trajectories after treatment. The patients expressed a desire for guidance in managing symptoms, stress, exercise, and nutrition to ease their “new normal” lives [19]. Recently, the importance of integrating oncological and supportive care has been increasing [20], as early interventions designed to provide supportive care for patients with cancer are in demand. Based on the findings of our qualitative interview, we hypothesized that an intervention introduced in the early stages of lung cancer treatment can help survivors return to their new normal lives.

Recognizing the critical need for early supportive interventions, we developed the Lung Cancer Comprehensive Care Program (LC^3^P). This program aims to support patients from diagnosis through their adjustment to posttreatment life, encompassing their return to work, family, and societal roles. This study aimed to evaluate the effectiveness of LC^3^P in enhancing psychological adjustment to cancer, with the hypothesis that participants receiving the intervention would demonstrate improved adjustment 6 months after surgery.

## Methods

### Study Setting

We designed a controlled before-and-after trial at the Samsung Comprehensive Cancer Center of the Samsung Medical Center (SMC), a university-affiliated hospital in Seoul, Republic of Korea, to evaluate the impact of LC^3^P compared to routine care. This design was selected to minimize the risk of information contamination between the control and intervention groups. This risk is particularly pertinent to educational interventions, in which the exchange of information between participants can confound outcomes.

### Eligibility Criteria

The inclusion criteria for the study are (1) age 18 years or older at enrollment, (2) diagnosis of stage I, II, or III NSCLC, and (3) a plan to undergo pulmonary resection. Patients with (1) recurrent lung cancer, (2) multiple sites of primary cancer, and (3) a history of other cancers or those who had received active cancer treatment within the past 3 years were excluded. Participants who canceled their pulmonary resection, received a confirmed diagnosis of stage IV lung cancer, or wished to withdraw from the study were excluded. All participants will be required to provide written informed consent before undergoing baseline assessment.

### Intervention

#### Developing the Intervention

The LC^3^P was developed following an extensive literature review [17,21,22], qualitative interviews [19], and cross-sectional studies [15]. These studies aimed to define the informational necessities and challenges faced by lung cancer survivors during their treatment journey. We discovered that the survivors adjusted their postcancer lives by reconciling their expectations with reality [19]. Physical symptoms, psychological challenges, and concerns regarding exercise and nutrition were also examined. Therefore, our intervention delivered timely and stage-specific information from the onset of treatment, primarily through educational videos. This format was selected due to its accessibility, patient friendliness, and potential to alleviate the workload of clinicians.

#### Content of the Intervention

The LC^3^P is a 2-part intervention. The first part of the program comprised 7 educational videos, 4 of which were designed to meet the specific needs of lung cancer survivors. The first video provided general information regarding lung cancer, treatment options, and postsurgery challenges such as pain, emphasizing the importance of smoking cessation and exercise. The second video covered postoperative recovery, addressed symptoms such as reduced lung function and fatigue and offered management strategies. The third video was an exercise tutorial for patients, and the fourth video offered nutritional guidance through a sample meal plan. The latter 3 videos provided general information regarding distress management, return to work [23], and fear of cancer recurrence. The second part of the program was the first postsurgical follow-up with a family medicine physician who assessed the patient’s unique needs using a study-specific checklist (Figure S1 in Multimedia Appendix 1). This visit focused on personalized care through counseling, medication management, and referrals to specialists as needed.

#### Delivery of the Intervention

Eligible participants received a brochure containing QR codes linked to educational videos (Figure S2 in Multimedia Appendix 1). We added QR codes for all the videos in a brochure and gave it to participants before surgery so they could preview the videos if they were curious about what to expect and what to work on after surgery. Participants were strongly encouraged to view the first video before surgery. The link to the first video was sent via SMS text message at the time of enrollment to increase patient accessibility. Around the time of discharge, a research assistant visited the intervention group participants to offer them a second copy of the brochure and sent links to each of the 3 videos, reminding them to watch the videos at their convenience. Data on compliance, satisfaction, and feedback on the educational videos were collected during follow-up visits. Measures were taken until the survivors reported watching all 4 videos or at their 1-year follow-up visit.

**Figure 1:**
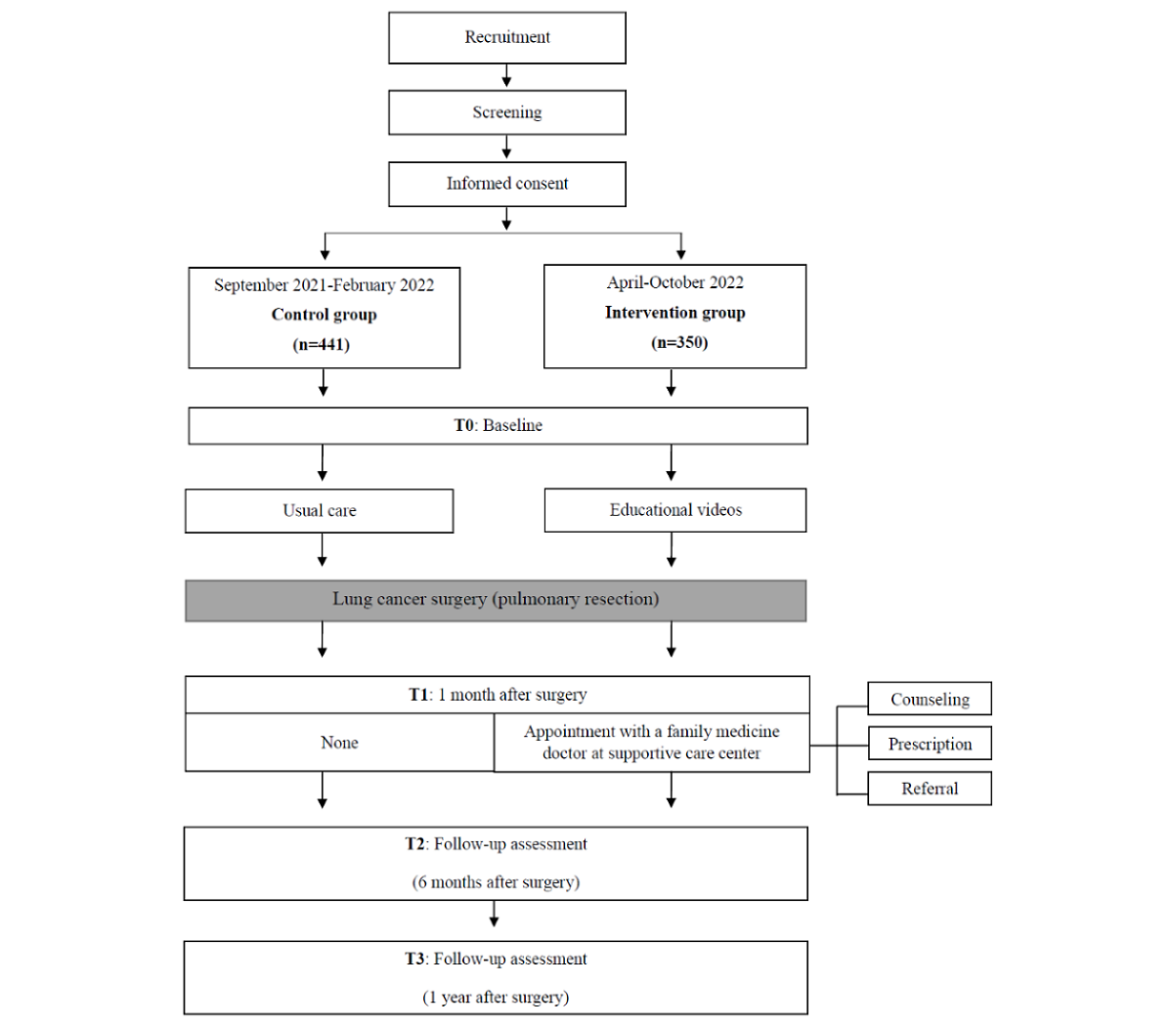
Flowchart of the Lung Cancer Comprehensive Care Program.

During the first follow-up visit after discharge, participants in the intervention group were scheduled to visit a family medicine doctor in addition to regular oncology appointments. During their appointment, the participants discussed any physical or psychological difficulties they had experienced after surgery, and the family medicine doctor provided tailored assistance based on the type and depth of their needs. As our intervention was educational, the participants in the intervention group were qualified to receive other concomitant care or additional necessary measures during the study. A flowchart of the study procedure and intervention is shown in Figure 1.

#### Control Group

Participants in the control group received routine preoperative and discharge information regarding hospital stay, costs, and postoperative care. They did not have access to educational programs or postoperative educational support.

### Outcomes

#### Primary Outcome Measures

The primary outcome of the study was psychological adjustment for cancer, measured 6 months after pulmonary resection. The Korean version of the Mini-Mental Adjustment to Cancer (Mini-MAC) was used to assess survivors’ psychological adjustments to cancer. The Mini-MAC is a 29-item scale scored on a 4-point Likert scale (1=definitely does not apply to me and 4=definitely applies to me) previously validated in Korea [24,25]. The Korean-validated scale consists of the following 4 dimensions: helpless-hopeless (8 items), anxious preoccupation (8 items), cognitive avoidance (4 items), and positive attitude (9 items). The positive attitude dimension contained items identical to those of fighting spirit and fatality from the original Mini-MAC. The total score is the sum of all dimension scores, with higher scores indicating a stronger use of coping strategies. Permission to use the Mini-MAC was obtained from the International Psycho-Oncology Society on May 19, 2021.

#### Secondary Outcome Measures

The secondary outcomes were unmet informational needs, quality of life, distress, and symptoms. Unmet informational needs were assessed using the information needs scale (3 items) from the Korean version of the Cancer Survivors’ Unmet Needs scale [26,27]. Health-related quality of life is measured using the Korean version of the European Organisation for Research and Treatment of Cancer Quality of Life Questionnaire—Core 30 [28]. Distress was assessed using the National Comprehensive Cancer Network Guidelines for Distress Management (version 2.2021) [29]. The European Organisation for Research and Treatment of Cancer Quality of Life Questionnaire—Lung Cancer 13 [30], the Chronic Obstructive Pulmonary Disease Assessment Test [31,32], and the modified Medical Research Council dyspnea scale [33] are used to assess the presence and severity of symptoms related to lung cancer.

The Korean version of the International Physical Activity Questionnaire-Short Form is used to identify participants’ levels of physical activity [34]. Depending on the level of activity derived from the questionnaire, patients were categorized into high, moderate, or low groups. The Hospital Anxiety and Depression Scale is a measure of depressive mood [35]. The components of each intervention and the hypothesized effects on the primary and secondary outcomes are shown in Figure S3 in Multimedia Appendix 1.

Sociodemographic variables such as age, sex, and level of education were acquired directly from the participants via self-reported questionnaires. Clinical characteristics, such as cancer stage, type of treatment, and postsurgical complications, were obtained from electronic medical records.

### Sample Size Calculation

Based on the primary hypothesis that the intervention group would demonstrate a 25% better psychological adjustment to cancer at 6 months after surgery than the control group, initial calculations with an effect size of 0.25 indicated that 252 participants per group would be required. We aimed for a sample size of approximately 300 per group to account for a dropout rate of 16%. However, to increase the robustness of our findings, allow for potential subgroup analyses, and ensure that the study had sufficient power to detect smaller effect sizes, we enrolled 400 participants per group. The sample size significantly exceeded the required number based on our initial estimates, thereby enhancing the statistical power and potential impact on the study's outcomes.

### Recruitment

Patients scheduled for pulmonary resection were recruited as controls to prevent contamination. The recruitment and study period for the control group were completed before the start of the intervention to ensure no overlap. Subsequently, recruitment to the intervention group began.

### Data Collection

**Figure 2:**
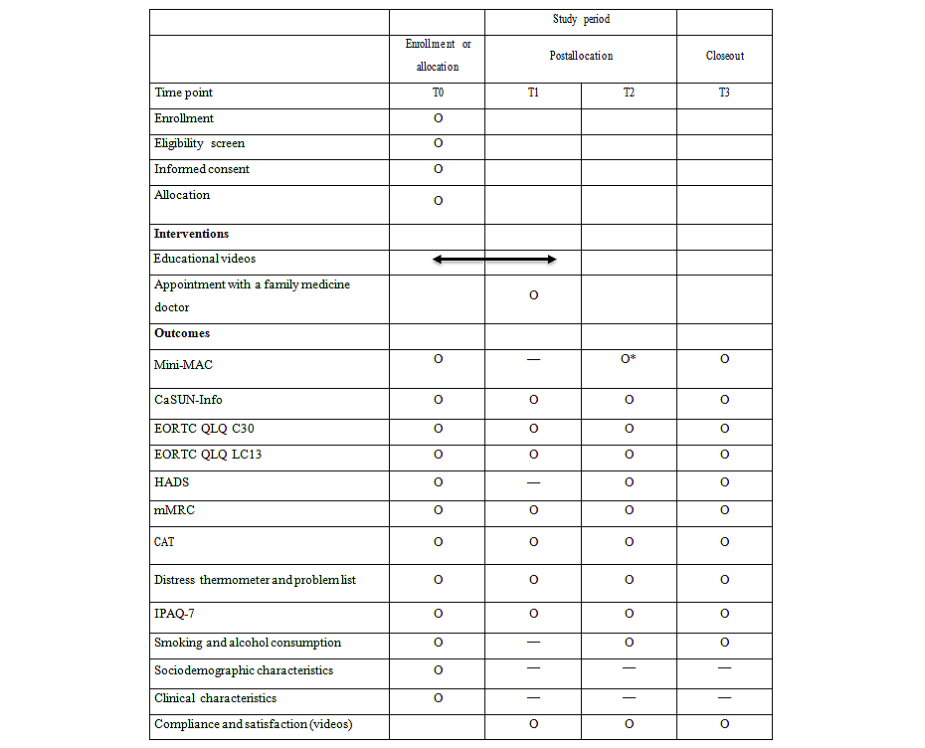
Schedule of enrollment, intervention, allocation, and assessments. CaSUN-Info: Cancer Survivors’ Unmet Needs Measure—Information domain; CAT: Chronic Obstructive Pulmonary Disease Assessment Test; EORTC QLQ C30: European Organisation for Research and Treatment of Cancer Quality of Life Questionnaire—Core 30; EORTC QLQ LC13: European Organisation for Research and Treatment of Cancer Quality of Life Questionnaire—Lung Cancer 13; HADS: Hospital Anxiety and Depression Scale; IPAQ-7: International Physical Activity Questionnaire-Short Form; Mini-MAC: Mini-Mental Adjustment to Cancer; mMRC: Modified Medical Research Council; T0 is baseline (before surgery); T1 is 1 month after surgery; T2 is 6 months after surgery; T3 is 1 year after surgery. *Primary outcome.

The clinical trial procedure and intervention are outlined in Figure 1, with the outcomes assessed at baseline (T0), 1 month (T1), 6 months (T2), and 1 year (T3) postoperatively. Baseline data will be collected before surgery and immediately after enrollment. To enhance participant retention and adherence, individuals in both groups received a small token of appreciation upon completion of each of the 3 postoperative follow-up assessments (T1-T3). For participants who discontinued or deviated from the intervention protocols, the data collected up to the point of departure were included in the intention-to-treat analysis to maintain the robustness of the trial results and minimize the impact of attrition bias. The data obtained from self-administered questionnaires were coded and stored in password-encrypted Microsoft Excel (Microsoft Corp) files on a secure computer server. The specific variables measured at each time point are listed in Figure 2.

### Data Monitoring

Although the intervention primarily comprised educational videos and consultations with a family medicine physician, the associated risk to the participants was anticipated to be minimal. Accordingly, the establishment of a formal data-monitoring committee was considered unnecessary. However, procedures for monitoring adverse events and their unintended effects remain in place. The research team regularly reviewed participants’ feedback and outcomes to identify potential concerns. Should any adverse events or unintended consequences arise, they will be addressed promptly, with details reported to the overseeing institutional review board (IRB) at the SMC, as per the standard protocol.

### Statistical Analysis

To assess the effectiveness of LC^3^P, we compared the outcomes between the intervention and control groups at multiple time points. The primary analyses involved independent sample 2-tailed *t* tests for continuous variables and chi-square tests for categorical variables to compare baseline characteristics and outcomes between the 2 groups at each follow-up. To account for repeated measures within participants over time and potential within-subject correlations, a mixed-model repeated-measures analysis was used.

In addition to primary analyses, sensitivity analyses probed the robustness of the trial findings under different assumptions regarding missing data and the effects of protocol adherence on treatment outcomes. This included intention-to-treat and per-protocol analyses. Moreover, subgroup analyses were conducted to examine the variability in treatment effects across different patient characteristics such as age, cancer stage, and the presence of comorbid conditions. All tests were 2-tailed, with a significance level of *P*<.05. To address multiple testing concerns, particularly with numerous secondary outcomes, we used Bonferroni correction or other suitable adjustments to preserve the overall type I error rate. All statistical analyses were conducted using STATA/MP (version 16.0; StataCorp).

### Ethical Considerations

This study was approved by the IRB of the SMC (SMC 2021-08-071). If any critical amendments, including but not limited to the eligibility criteria, outcomes, and analyses, become necessary in the future, modifications to the protocol will be shared with all relevant parties, including all investigators, the IRB committee, and the trial registry. A trained research assistant initiated contact with potential participants after screening for eligibility. Written informed consent forms were obtained after the purpose, procedure, and potential harm of participating in the study were explained. Informed consent was obtained from each patient before enrollment in the study. Personal data such as sociodemographic information, clinical characteristics, and outcomes measured at each visit were coded with a unique study identification number in password-encrypted Microsoft Excel files stored on a secure computer server to prevent unauthorized access. Hard copies of the questionnaire were retained for a decade after the completion of the study. Both the primary investigator and coinvestigators had access to the final data set without any contractual regulations.

## Results

The historical control group (n=441) was recruited from September 8, 2021, to April 20, 2022, and the intervention group (n=350) was recruited from April 22, 2022, to October 17, 2022. All statistical analyses will be performed upon completion of the study.

As part of a nationally funded study, the results and details of the procedures will be made publicly available upon completion. The findings of this study will be actively shared through publications in peer-reviewed academic journals and at national and international scientific conferences. Regarding the intervention, the content and access to the educational videos will be refined based on the feedback patients provide on their first follow-up visit and will be made available on YouTube and to other health care professionals within the same health care institution, including oncologists and oncology nurses. The full protocol for this study is currently available at the ClinicalTrials.gov (NCT05078918). The raw data for this study will be shared upon request.

## Discussion

### Principal Findings

In this controlled before-and-after trial, the effectiveness of LC^3^P in enhancing psychological adjustment to lung cancer treatment was examined. The LC^3^P distinguishes itself by offering informational and personalized support from the initial phases of treatment.

### Comparison to Prior Work

Lung cancer is often diagnosed during screening examinations or in the absence of a symptomatic presentation, resulting in a broad spectrum of patient effects upon diagnosis. Unlike previous interventions that typically introduced posttreatment survivorship care, our approach provided support from the outset. The median time for lung cancer survivors to engage in OncoLife and the LIVESTRONG Care Plans, 2 web-based programs providing survivorship care plans, was 1 year after treatment [11]. Similar trends in delivering survivorship care plans at the end of treatment have been reported, with the earliest delivery being within a year [36]. While providing survivorship care at the end of treatment allows survivors to process information relevant to their cancer continuum, some findings suggest that patients want to receive survivorship care plans during earlier stages of treatment [37]. The early engagement of the LC^3^P aligns with emerging preferences for immediate survivorship care, as evidenced by patients expressing a desire to receive such information at earlier stages of their treatment trajectory.

In addition to early education, the intervention included general and tailored support. The educational videos offered to patients prior to treatment consisted of general information about the disease and survivorship care and were therefore applicable to all patients with NSCLC who are at stage I to III and who are planning to undergo lung resection. The second half of the intervention is personalized for each patient, as patients discuss their individual needs with their primary care physicians to determine the appropriate intervention to address their concerns. By taking a holistic approach to addressing patients’ general and individual concerns about their disease, we expect lung cancer survivors to have a better understanding of their disease and to better adjust by recognizing and accepting the differences between their lives before and after cancer. In addition, the relative simplicity of LC^3^P allows for its easy dissemination in routine care. Translating empirical evidence into clinical practice is difficult for several reasons including feasibility and pragmatism [38]. The educational videos that are part of our intervention are available on a web-based, easily accessible platform (eg, YouTube), allowing patients to obtain the information they need at their convenience. Displaying a booklet of QR codes linked to educational videos in outpatient clinics or inpatient wards is another viable option that does not interfere with the primary responsibilities of health care professionals. Furthermore, primary care and family medicine physicians commonly manage the long-term care of cancer survivors [39,40]. Given these considerations, we believe integrating the LC^3^P into the routine care of patients with cancer will have fewer barriers.

### Limitations

Despite these advantages, this study has some limitations that must be addressed. First, the design of the intervention might be more appealing to participants who are actively seeking ways to adapt to life after cancer, as opposed to those who are less motivated or indifferent about making such adjustments. In addition, although the use of a historical comparison group was intended to minimize the impact of exposure to our intervention in patients with lung cancer, the limitations of using a nonparallel comparison group should not be ignored. Second, socioeconomic and geographic disparities among older patients with cancer could affect accessibility to web-based educational videos. Although disseminating educational videos through web-based platforms allows cancer survivors to access as much information as they need at their convenience, it can be difficult for survivors with limited access to technology to access this information. If found to be promising, future studies should address the disparities in accessing web-based educational videos. Finally, the trial was registered after the recruitment commenced. However, there were no changes in the study design, measurement, or analysis methods after receiving the initial ethics approval.

In conclusion, the LC^3^P represents a proactive, holistic prehabilitation intervention aimed at supporting lung cancer survivors, as they navigate the transition to life after treatment. Our findings highlight the impact of early informational support on patients’ psychological adaptation and postdiagnosis stress management. This could potentially establish a new standard for the timing and delivery of survivorship care, emphasizing the benefits of early intervention.

## Appendix

Multimedia Appendix 1 Supplementary Figures 1-3.

## Abbreviations

IRB: institutional review board
LC3P: Lung Cancer Comprehensive Care Program
Mini-MAC: Mini-Mental Adjustment to Cancer
NSCLC: non–small cell lung cancer
SMC: Samsung Medical Center

## Data Availability

The data sets generated and analyzed during this study are available from the corresponding author on reasonable request.
