# Supplementary material for: Supporting Life Adjustment in Patients With Lung Cancer Through a Comprehensive Care Program: Protocol for a Controlled Before-and-After Trial
Source: JMIR Res Protoc. 2024 Feb 13;13:e54707. doi: 10.2196/54707 (PMC10900087; doi:10.2196/54707)
Supplement: Multimedia Appendix 1 [file resprot_v13i1e54707_app1.docx]

**Figure S1**. Checklist used for the intervention group’s appointment with the Family Medicine doctor.


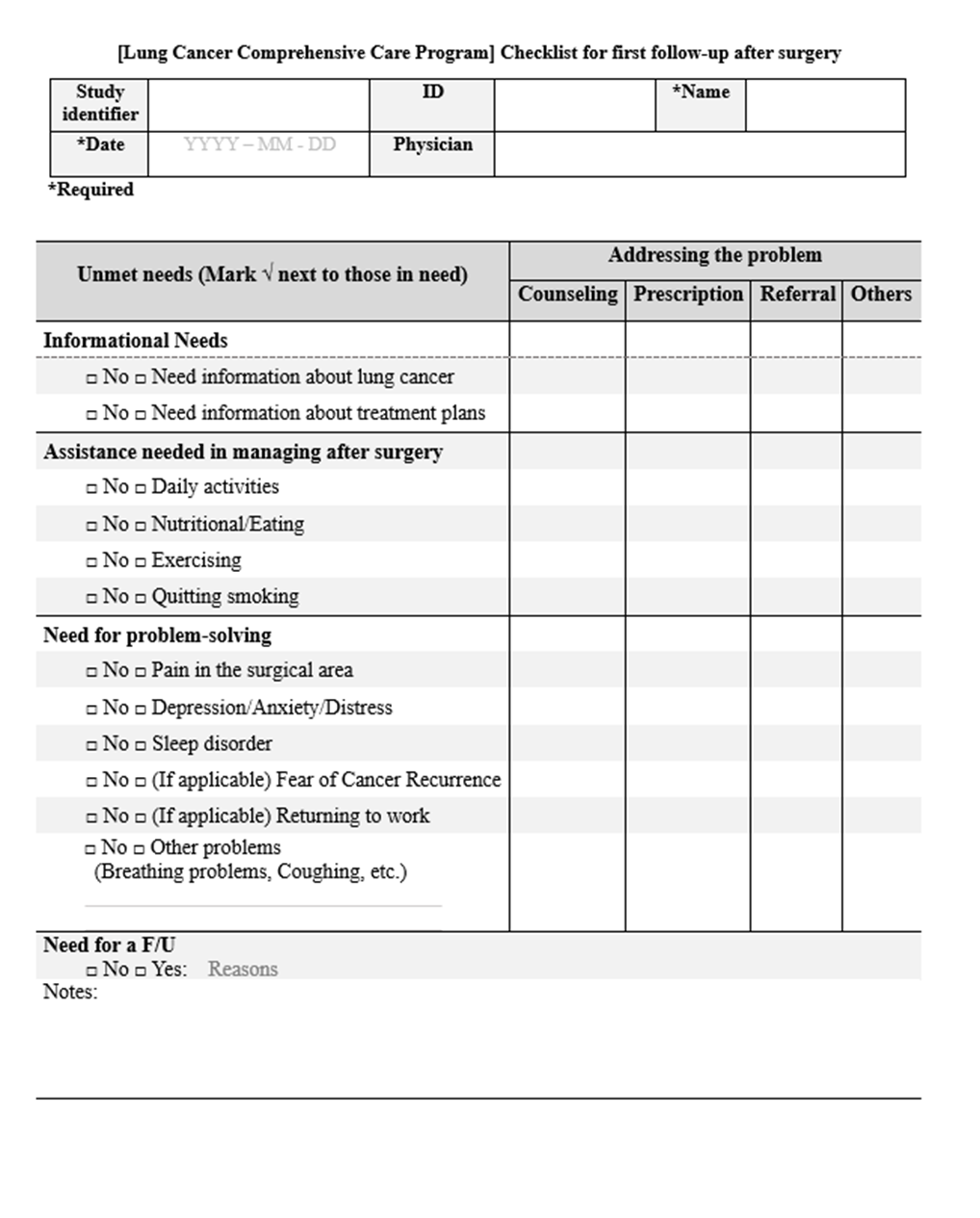


**Figure S2**. Screenshots of the 7 educational videos provided to the intervention group.


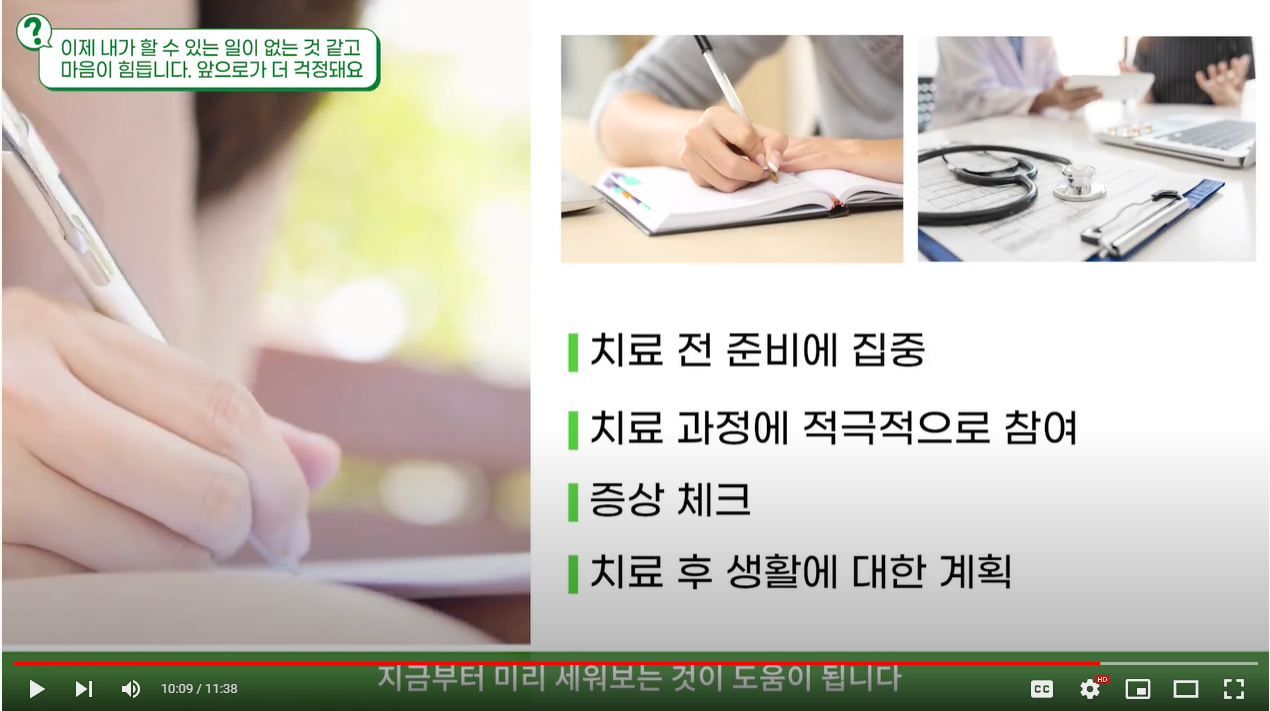

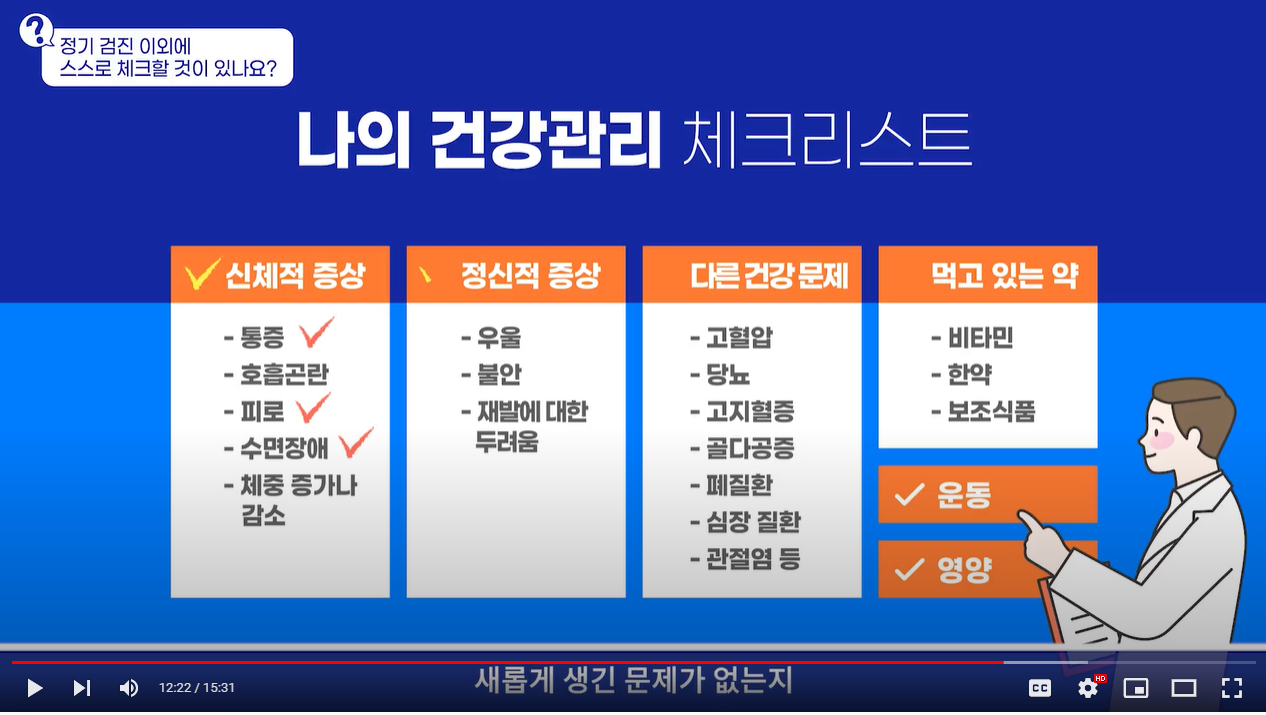


#2. How can I recover faster after surgery?

#1. How should I prepare for surgery?


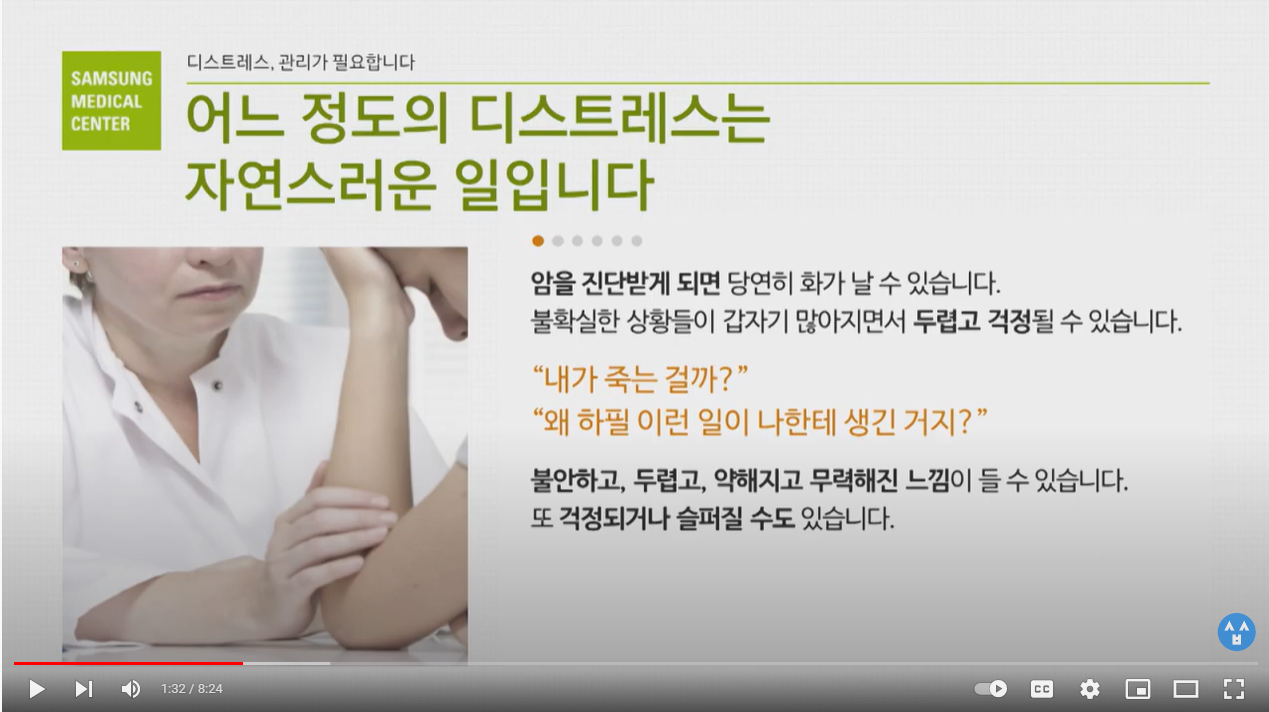


#5. Managing Distress

#6. Returning to Work


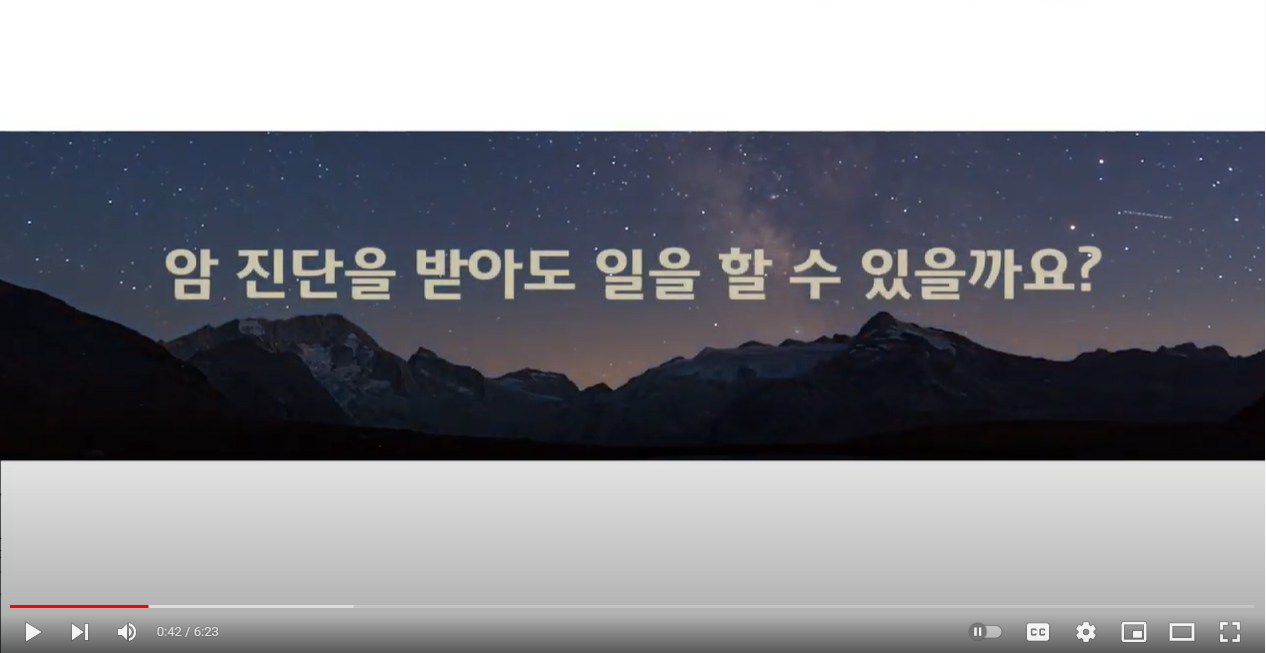


#7. Fear of Cancer Recurrence


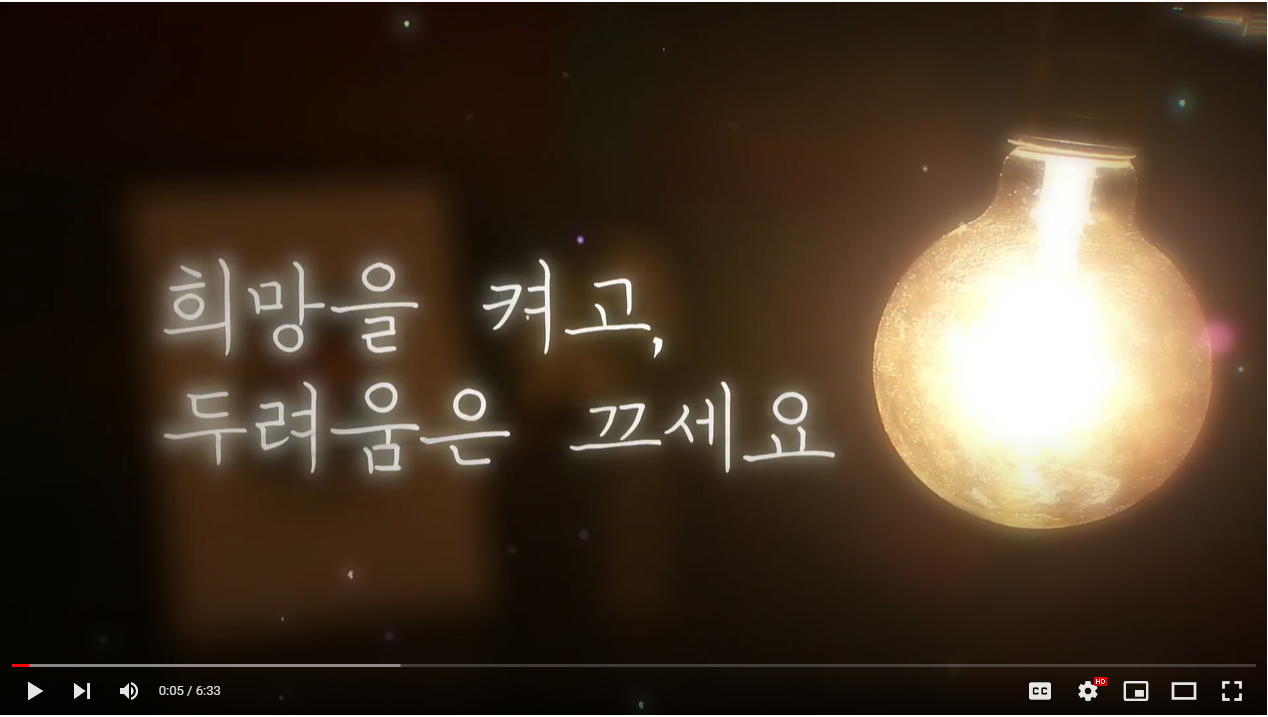

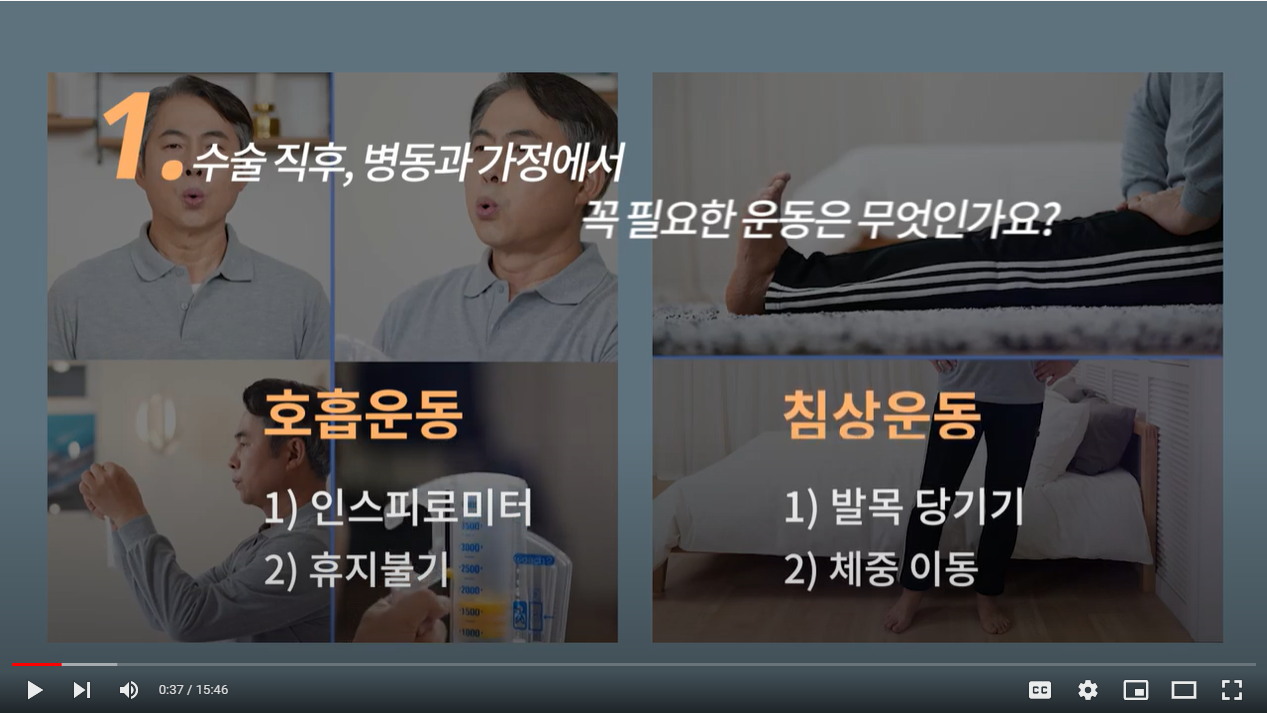


#3. What exercises should I do after surgery?

#4. How should I eat after surgery?


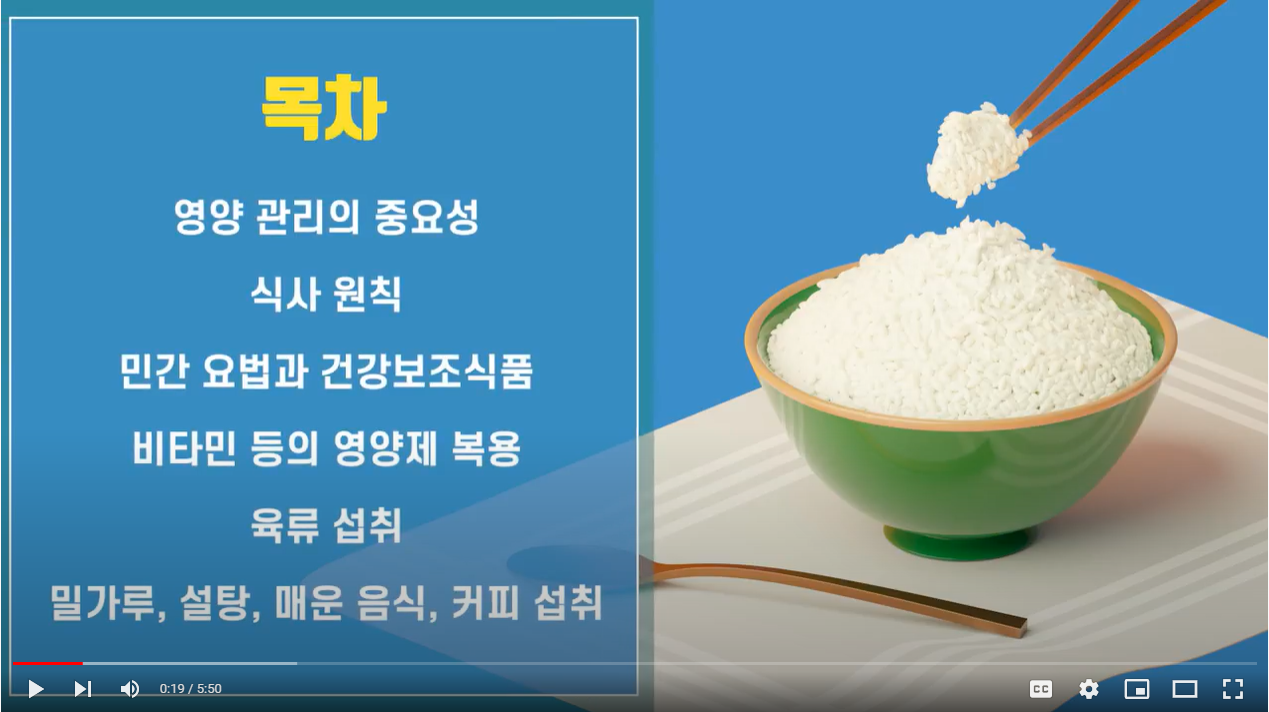


**Figure S3.** The components of each intervention and the hypothesized effects on primary and secondary outcomes.

**CaSUN-Info**

**Mimi-MAC**

**HADS**

**Video 1: Lung cancer treatment**

**Video 2: Postop recovery**

**Video 3: Exercise**

**Video 4: Nutrition**

**Video 5: Distress**

**Video 6: Return to work**

**Video 7: Fear of cancer recurrence**

**Supportive Care Physician visit**

**Smoking/Alcohol**

**mMRC, CAT, IPAQ**

**EORTC QLQ C30**

**EORTC QLQ LC13**

**Intervention components**

**Outcomes measurement**
